# Exploring Correlates of Resource Insecurity Among Older Black or African Americans with HIV in Ohio

**DOI:** 10.1007/s40615-024-02158-y

**Published:** 2024-09-04

**Authors:** Yanil V. Ramirez, Gisella M. Drouet Saltos, Timothy N. Crawford

**Affiliations:** 1 Wright State University Boonshoft School of Medicine, Dayton, OH USA; 2 Wright State University Boonshoft School of Medicine, Population and Public Health Sciences, Dayton, OH USA; 3 Family Medicine, Wright State University Boonshoft School of Medicine, Dayton, OH USA

**Keywords:** HIV, People with HIV, Resource insecurity, Discrimination, Stigma, Substance use

## Abstract

**Objectives:**

Resource insecurity is a social determinant of health that can impact people with HIV (PWH), in particular older African Americans (AA) or blacks with HIV. The purpose of this study was to identify resource insecurities among older Blacks or AA PWH specifically related to food and housing. Secondary focus was to find associations between resource insecurity and substance use history, stigma, and various forms of discrimination.

**Methods:**

Eligible participants (*N* = 52) of this cross-sectional study were 50 years old or older, identified as Black or AA, diagnosed with HIV, and living in Ohio. Food insecurity was assessed using the Household Food Insecurity Access Scale and housing insecurity was defined as not having stable housing. Resource insecurity was categorized into food and housing secure, food or housing insecure, and food and housing insecure.

**Results:**

Almost half (48.1%) of participants reported housing insecurity, with approximately 58.0% experiencing food insecurity, and 38.5% facing both. Current substance use, particularly opiates, showed significant association with resource insecurity (OR = 5.54; 95% CI = 1.91–17.30). Moreover, experiences of everyday (OR = 1.19; 95% CI = 1.10–1.30) or major forms (OR = 1.75; 95% CI = 1.33–2.39) of discrimination, as well as HIV stigma (OR = 1.24; 95% CI = 1.01–1.55), were also linked to increased odds of resource insecurity among participants.

**Conclusions:**

Findings highlight how social factors contribute to resource insecurity among older AA PWH. Understanding the factors offers insight for targeted intervention in the fight against HIV transmission.

**Supplementary Information:**

The online version contains supplementary material available at 10.1007/s40615-024-02158-y.

## Introduction

Scientific and medical advancements in HIV have contributed to earlier detection, reduced transmissions, and prolonged life expectancy in the USA [1, 2]. Nonetheless, HIV disproportionately affects racial minorities, particularly Black or African Americans (AA) [2]. Over 50% of People with HIV (PWH) are ages 50 years and up (considered older adults) in the USA and older adults make up 17% of all new HIV cases [1, 3]. African Americans make up 42% of new diagnoses among older adults and comprise 39% of all older PWH [3]. Addressing HIV disparities is crucial in ensuring equitable access to prevention, care, and support services, while fostering a healthier and more inclusive society.

Social determinants of health encompass the conditions in which individuals are born, grow, live, work, and age [4]. These factors, including social, economic, and environmental elements, significantly impact an individual’s overall well-being. Moreover, disparities in social determinants contribute to health inequities, creating greater challenges and poorer outcomes for certain populations compared to others. PWH are particularly affected by issues such as food and housing insecurity [5, 6]. This is because this population is likely to experience resource insecurity due to health-related challenges, stigma and discrimination, limited social support, high medical costs—all of which impact their ability to work and earn stable income [7]. Resource insecurity (food and housing insecurity) has been identified as a challenge to achieving the National HIV/AIDS Strategy goal of improving HIV-related health outcomes of PWH [8].

Researchers have shown resource insecurity to be associated with risky sexual behaviors, lower CD4 counts, unsuppressed viral loads, and overall poorer physical and mental health among PWH [9–12]. Whittle et al. [10] conducted a survey involving 34 PWH in the San Francisco Bay Area to explore the correlation between food insecurity and engaging in risky sexual behaviors. The group found food insecurity to be a strong contributor to risky sexual behaviors. Additionally, a systematic review encompassing various studies from different countries highlighted a pattern of low adherence to antiretroviral therapy (ART) among women and children living with HIV who experienced food insecurity. The authors characterized this phenomenon as a detrimental cycle wherein PWH faced obstacles in adhering to ART due to concerns about taking medications on an empty stomach, apprehensions regarding potential side effects in the absence of proper nutrition, or the need to exchange ART for food [11].

Moreover, housing insecurity has been identified as another factor adversely affecting ART adherence. A survey conducted in Atlanta, GA, USA, with HIV-positive men and women addressed issues related to housing, food, and transportation access. The lack of essential resources was found to have a negative impact on adherence, with this effect being indirectly influenced by a dearth of access to social support and services [12].

Additionally, resource insecurity can exacerbate the stigma and discrimination faced by PWH [13]. Furthermore, individuals from minority races also experience the impact of social determinants on their health. Addressing these factors is crucial for promoting health equity and improving overall outcomes.

Numerous studies emphasize the complex interplay between PWH, resource insecurity, discrimination, and substance use. Racial discrimination has been found to significantly impact HIV-related stigma, depression, and social support, whereas depression and social support have been directly impacted by HIV-related stigma and housing insecurity [14]. Previous literature further demonstrates that food insecurity and substance use can increase likelihood of engaging in risky sexual behavior that ultimately increases risk of contracting HIV [15, 16]. A study in Russia observed a strong association between PWH that experience severe food insecurity and needle sharing, thus, increasing HIV transmission risk [5]. Moreso, Whittle et al. [17] found a strong association between very low food security and higher odds of substance use among women with HIV. However, little research has been conducted understanding factors associated with resource insecurity among older PWH, specifically AAs.

Abundant literature highlights the impact of resource insecurity on HIV care. For instance, studies have delved into the effects of food insecurity on medication adherence [18]. Others have examined the relationship between characteristics of food insecurity and internalized stigma in women living with HIV [19]. The intersection of homelessness and ART outcomes have also been explored [20]. Despite these investigations, there are notable gaps in the literature. There is a dearth of research understanding resource insecurity and factors that are associated with resource insecurity among older PWH, specifically older Black PWH. With the growing population of older PWH, the continued disproportionate impact of HIV on AAs, persistent HIV-stigma, and the influence of social determinants of health on access to care, understanding factors that are associated with resource insecurity and how resource insecurity affects the care of this population becomes increasingly crucial.

In the USA, there has been a recent increase in the percentage of households with food insecurity at some time during the year (10.5% in 2020 to 12.8% in 2022). This increase was also experienced among older adults (defined as 65 years of age and older) living alone, with 11.4% being food insecure in 2022—an increase from 8.3 percent in 2020 [21]. Approximately half of single unhoused adults in the US are over the age of 50 [22]. Furthermore, as individuals age, their ability to perform daily activities diminishes, often leading to changes in their housing situation. Additionally, their financial circumstances may change, resulting in the inability to afford facilities that could aid them [23, 24]. Tong et al. [25] conducted a cross-sectional study analyzing factors associated with food insecurity in a group of 350 unhoused adults 50 years of age and older. The study revealed that older unhoused individuals are at greater risk of experiencing food insecurity. PWH who are over 50 years of age are already a vulnerable population due to both the symptoms of aging and HIV. Other studies have shown that food and housing insecurity in older individuals have been associated with poor HIV care engagement (not adhering to antiretroviral medications, not maintaining sustained viral suppression, and risk of forward transmission) [6, 26].

This descriptive, exploratory study sought to fill existing gaps by thoroughly exploring resource insecurity, specifically related to food and housing, among a predominant sample of older Black or AA men living with HIV in Ohio. We focused on older individuals because not only do they have HIV but are also aging, experiencing the compounded effects of HIV, geriatric-related symptoms, and food and housing insecurity, all likely influencing health outcomes in this population. The primary focus was to identify resource insecurities within the study population, and to explore what demographic and social and behavioral factors were associated with resource insecurity. The choice of Ohio as the study location is significant due to its relevance in the context of the Ending the HIV Epidemic initiative. Three counties in Ohio—Cuyahoga, Hamilton, and Franklin—were designated as focal points, along with 52 other US counties, for the initiative, as these counties accounted for over half of all new infections in 2019 [27]. Therefore, understanding the resource insecurities within this population in Ohio can provide valuable insights for targeted interventions and support in the ongoing efforts to combat HIV transmission.

## Methods

### Study Site and Participants

This was a pilot study focused on examining the relationships among stigma, discrimination, and HIV care outcomes among older AA PWH [27]. Participants were recruited to participate in an online survey in the fall of 2021 that captured data on demographics, HIV care engagement, experiences of stigma and discrimination, and other social determinants of health. Participants were recruited through the social media Facebook page of Equitas Health Institute, one of the leading HIV care providers in Central and Southwestern Ohio. Participants were eligible to complete the survey if they were diagnosed with HIV, living in Ohio at the time of the survey, able to read English, 50 years of age and older, and identified as Black or AA. Participants received a $25 gift card upon completion of the survey. The study was approved by Wright State University’s Institutional Review Board.

### Study Measures

Demographic characteristics were self-reported and included age, years with HIV, gender, sexual orientation, education, employment status, yearly income, history of injection drug use, history of being unhoused, and history of incarceration.

Current substance use was assessed using the National Institute on Drug Abuse modified Alcohol, Smoking, and Substance Involvement Screening Test (NIDA-Assist) [28]. Participants were asked about their lifetime use (“in your life, which of the following substances have you ever used”) and their past 3 months use (in the past 3 months, how often have you used the substances you mentioned). In the tool, participants are asked to report non-medical use of tobacco products (cigarettes, chewing tobacco, cigars, etc.), alcoholic beverages (beer, wine, spirits, etc.), cannabis (marijuana, pot, grass, hash, etc.), cocaine (coke, crack, etc.), amphetamine type stimulants (speed, diet pills, ecstasy, etc.), inhalants (nitrous, glue, petrol, paint thinner, etc.), sedatives or sleeping pills (Valium, Serepax, Rohypnol, etc.), Hallucinogens (LSD, acid, mushrooms, PCP, Special K, etc.), Opioids (heroin, morphine, methadone, codeine, etc.), and other. A participant that reported using any substance at least once in the past 3 months was considered a current user. The substances that were of focus for this study were cannabis, cocaine, amphetamine type stimulants, and opioids.

Risky alcohol use was assessed using the 3-item Alcohol Use Disorder Identification Test Consumption screening questionnaire (AUDIT-C) to identify persons with risky alcohol use. The AUDIT-C is the modified versions of the original 10-item instrument. The AUDIT-C consists of the following questions: “How often do you have a drink containing alcohol,” “How many standard drinks containing alcohol do you have on a typical day,” and “How often do you have six or more drinks on one occasion.” A summed scale score ranging from 0 to 12 was created. Risky alcohol use was defined as a summed score ≥ 4 for men and ≥ 3 for women [29].

HIV-related stigma was assessed using the Stigma Revised Scale. This is a 10-item shortened version of the Berger HIV Stigma 40-item scale. Item responses were on a 4-point scale and ranged from “strongly disagree” to “strongly agree.” An overall stigma score was created by summing up the 10-items. Scores ranged from 10 to 40 with higher scores denoting greater experiences of stigma. The Stigma Revised Scale has four subscales which include personalized stigma (range = 3 to 12), disclosure concerns (range = 2 to 8), negative self-image (range = 3 to 12), and public attitudes regarding HIV (range = 2 to 8) [30].

Everyday discrimination was assessed using the Everyday Discrimination Scale which is a nine-item scale which measures chronic and routine unfair treatment in everyday life. Participants were asked to list how often any of the experiences (e.g., “You are treated with less courtesy than other people are,” “You are treated with less respect than other people are,” “People act as if they are afraid of you”) happened to them in their day-to-day life. Item responses ranged from “almost every day” to “never,” and were on a 5-point scale (0–5). The overall score ranged from 0 to 45 with higher scores denoting more experiences with everyday discrimination [31].

Major forms of discrimination assessed using the major experiences of discrimination scale which is a nine-item scale that assesses lifetime experiences of discrimination. Participants were asked if there was ever a time when they were unfairly treated (e.g., “At any time in your life, have you ever been unfairly fired?”, “For unfair reasons, have you ever not been hired for a job?”). Participants could select “yes” (1) or “no” (0). A summed score was created with a range of 0 to 9 with higher scores representing more lifetime experiences of discrimination [32].

Food insecurity assessed using the Household Food Insecurity Access Scale (HFIAS). This 18-item questionnaire asks about the occurrence of food insecurity (e.g., in the past four weeks, did you worry that your household would not have enough food?) and frequency of endorsed occurrence (i.e., how often did this happen?—rarely, sometimes, or often). Frequency and occurrence questions were merged (i.e., those who said no to occurrence were considered “never” in frequency) to create 9-items on a 3-point scale, ranging from 0 = never to 3 = often. The food insecurity scale score was created by summing up the merged nine items. The score ranges from 0 to 27 with higher scores denoting more food insecurity [33]. Additionally, we dichotomized food insecurity into food secure (score = 0) and food insecure (score > 0).

Housing insecurity assessed using two questions: “Which of the following best describes the residence in which you currently live?” and “Given your total household income, how difficult is it to meet your monthly housing costs including rent/mortgage, property taxes, and utilities” (very easy, fairly easy, neutral, fairly difficult, and very difficult). Participants were considered to be housing insecure if they responded that their residence was a self-contained room with or without amenities (not in an apartment or house with other people), outdoors/on the street/parks/in a car, couch surfing, transition house/halfway house/safe house, shelter, or jail OR they reported that it was fairly difficult or very difficult to meet monthly housing costs [34].

Resource insecurity was defined by grouping housing and food insecurity into an ordinal level variable. The categories were neither food or housing insecurity, food or housing insecurity, and both food and housing insecurity.

The full questionnaire may be found in online resource 1.

### Statistical Analysis

The study sample was analyzed using descriptive statistics. Means and standard deviations were used for continuous variables; frequencies and percentages were used for categorical variables. To explore differences in resource insecurity by demographic and social variables, chi-square tests and ANOVAs were conducted. Additionally, simple ordinal logistic regressions were conducted to obtain crude odds ratios (OR) and 95% confidence intervals (CI). All data were analyzed using R version 4.1 and *p*-values < 0.05 were regarded as statistically significant.

## Results

Table 1 presents the descriptive statistics for the study participants. The average age of the study pariticipants was 53.7 ± 2.1 years (range = 50 to 58) and the average number of years since HIV diagnosis was 2.9 ± 1.7 years. The majority of the study participants were male (94.2%), had a college degree or higher (59.6%), and identified as heterosexual (63.5%). Almost half of the study participants had a history of incarceration (44.2%) and injection drug use (48.1%). Substance use was highly prevalent among the participants with 53.8% and 50.0% of the sample self-reporting use of cocaine and opiate use in the past 3 months, respectively. The mean HIV stigma scale was 29.5 ± 2.7 and the mean everyday discrimination scale was 24.6 ± 7.9. Additionally, approximately 29.0% of the sample were either food or housing insecure and approximately 39.0% were food and housing insecure.

**Table 1:** Descriptive statistics among older African Americans living with HIV in Ohio (*N* = 52)

Variable	Total *n* (%)
Age – mean (sd)	53.65 (2.06)
Years with HIV	2.87 (1.74)
Education	
HS degree or less	17 (32.7)
Technical school/some college	4 (7.7)
College degree or higher	31 (59.6)
Employment status	
Full time	17 (32.7)
Part time	21 (40.4)
Retired	14 (26.9)
Yearly income	
< $25,000	31 (59.6)
≥ $25,000	21 (40.4)
Gender	
Female	3 (5.8)
Male	49 (94.2)
Sexual orientation	
Heterosexual	33 (63.5)
Sexual minority	19 (36.5)
History of injection drug use	
No	27 (51.9)
Yes	25 (48.1)
History of being unhoused	
No	31 (60.8)
Yes	20 (39.2)
History of incarceration	
No	29 (55.8)
Yes	23 (44.2)
Cocaine use in the past 3 months	
No	24 (46.2)
Yes	28 (53.8)
Opiate use in the past 3 months	
No	26 (50.0)
Yes	26 (50.0)
Cannabis use in the past 3 months	
No	17 (32.7)
Yes	35 (67.3)
Amphetamine use in past 3 months	
No	25 (48.1)
Yes	27 (51.9)
Risky alcohol use	
No	8 (15.4)
Yes	44 (84.6)
Overall HIV stigma scale	29.54 (2.73)
Personalized stigma (enacted stigma)	9.17 (0.94)
Disclosure (anticipated stigma)	5.79 (0.75)
Negative self-image (internalized stigma)	8.52 (1.55)
Public attitudes (anticipated stigma)	6.06 (1.06)
Everyday discrimination scale	24.56 (7.94)
Major forms of discrimination scale	6.52 (2.12)
Housing insecurity	
No	27 (51.9)
Yes	25 (48.1)
Food insecurity	
No	22 (42.3)
Yes	30 (57.7)
Resource insecurity	
Neither	17 (32.7)
Food or housing	15 (28.8)
Food and housing	20 (38.5)

Table 2 presents the statistics by the three resource insecurity categories. Participants who were both food and housing insecure were more likely to have a history of injection drug use compared to those who were food or housing insecure and those with neither food or housing insecure (70.0% versus 53.3% versus 17.6%). Those with food and housing insecurity were more likely to have a history of incarceration compared to the other resource insecurity categories (60.0% versus 53.3% versus 17.6%). Similar associations were observed among those who self-reported current use of cocaine, opiates, cannabis, and amphetamines. Overall HIV stigma and anticipated stigma were associated with resource insecurity with those with food and housing insecurity having higher mean scores compared to the other resource insecurity categories. This was also similar for everyday and major forms of discrimination.

**Table 2:** Characteristics by food and housing insecurity among older people living with HIV (*N* = 52)

Variable	All participants *n* (%)	Neither *n* (%)	Food or housing *n* (%)	Food and housing *n* (%)	*p*-value
Total	52 (100.0)				
Age – mean (sd)	53.65 (2.06)	54.06 (1.85)	52.00 (1.25)	54.55 (2.04)	< 0.001
Years living with HIV – mean (sd)	2.87 (1.74)	3.00 (1.70)	2.67 (1.54)	2.90 (1.97)	0.8
Education					0.004
HS degree or less	17 (32.7)	3 (17.6)	10 (66.7)	4 (20.0)	
Technical school or higher	35 (67.3)	14 (82.4)	5 (33.3)	16 (80.0)	
Employment status					< 0.001
Full time	17 (32.7)	0 (0.0)	5 (33.3)	12 (60.0)	
Part time	21 (40.4)	5 (29.4)	9 (60.0)	7 (35.0)	
Retired	14 (26.9)	12 (70.6)	1 (6.7)	1 (5.0)	
Yearly income					0.2
< $25,000	31 (59.6)	9 (52.9)	7 (46.7)	15 (75.0)	
≥ $25,000	21 (40.4)	8 (47.1)	8 (53.3)	5 (25.0)	
Gender					0.3
Female	3 (5.8)	2 (11.8)	1 (6.7)	0 (0.0)	
Male	49 (94.2)	15 (88.2)	14 (93.3)	20 (100.0)	
Sexual orientation					< 0.001
Heterosexual	33 (63.5)	17 (100.0)	9 (60.0)	7 (35.0)	
Sexual minority	19 (36.5)	0 (0.0)	6 (40.0)	13 (65.0)	
History of injection drug use					0.006
No	27 (51.9)	14 (82.4)	7 (46.7)	6 (30.0)	
Yes	25 (48.1)	3 (17.6)	8 (53.3)	14 (70.0)	
History of being unhoused					0.12
No	31 (60.8)	12 (75.0)	6 (40.0)	13 (65.0)	
Yes	20 (39.2)	4 (25.0)	9 (60.0)	7 (35.0)	
History of incarceration					0.025
No	29 (55.8)	14 (82.4)	7 (46.7)	8 (40.0)	
Yes	23 (44.2)	3 (17.6)	8 (53.3)	12 (60.0)	
Cocaine use in the past 3 months					0.001
No	24 (46.2)	14 (82.4)	5 (33.3)	5 (25.0)	
Yes	28 (53.8)	3 (17.6)	10 (66.7)	15 (75.0)	
Opiate use in the past 3 months					0.004
No	26 (50.0)	14 (82.4)	6 (40.0)	6 (30.0)	
Yes	26 (50.0)	3 (17.6)	9 (60.0)	14 (70.0)	
Cannabis use in the past 3 months					< 0.001
No	17 (32.7)	12 (70.6)	3 (20.0)	2 (10.0)	
Yes	35 (67.3)	5 (29.4)	12 (80.0)	18 (90.0)	
Amphetamine use in past 3 months					0.002
No	25 (48.1)	14 (82.4)	6 (40.0)	5 (25.0)	
Yes	27 (51.9)	3 (17.6)	9 (60.0)	15 (75.0)	
Risky alcohol use					0.034
No	8 (15.4)	6 (35.3)	1 (6.7)	1 (5.0)	
Yes	44 (84.6)	11 (64.7)	14 (93.3)	19 (95.0)	
Overall HIV stigma scale – mean (sd)	29.54 (2.73)	28.47 (2.79)	29.73 (2.52)	30.30 (2.68)	0.050
Personalized stigma (enacted stigma) – mean (sd)	9.17 (0.94)	8.71 (0.99)	9.20 (1.01)	9.55 (0.69)	0.007
Disclosure (anticipated stigma) – mean (sd)	5.79 (0.75)	5.65 (0.61)	5.87 (0.64)	5.85 (0.93)	0.40
Negative self-image (internalized stigma) – mean (sd)	8.52 (1.55)	8.65 (1.11)	8.73 (1.16)	8.25 (2.07)	0.90
Public attitudes (anticipated stigma) – mean (sd)	6.06 (1.06)	5.47 (0.87)	5.93 (1.03)	6.65 (0.93)	0.003
Everyday discrimination scale – mean (sd)	24.56 (7.94)	17.82 (7.74)	26.40 (7.82)	28.90 (3.26)	< 0.001
Major forms of discrimination scale – mean (sd)	6.52 (2.12)	4.88 (1.96)	6.87 (2.03)	7.65 (1.39)	< 0.001

Bolded numbers denote significant results at the .05 level

Ordinal logistic regressions were conducted to explore the crude associations between the demographic and social factors and resource insecurity. The odd ratios were interpreted as the odds of resource insecurity (food and housing or food or housing versus neither). Sexual orientation was associated with resource insecurity with sexual minorities have an 11-fold (OR = 11.64; 95% CI: 3.55–43.92) increase in odds of resource insecurity compared to heterosexuals. For participants with a history of injection drug use, the odds of resource insecurity were 5.62 (95% CI: 1.93–17.62) times that of those without a history of injection drug use. For those with a history of incarceration, the odds of resource insecurity were 3.85 (95% CI: 1.36–11.53) times that of those without a history of incarceration. For current substance use, the odds of resource insecurity were higher for those who self-reported cocaine use (OR = 6.92; 95% CI: 2.31–22.60), opiate use (OR = 5.54; 95% CI: 1.91–17.30), cannabis use (OR = 12.09; 95% CI: 3.49–49.15), and amphetamine use (OR = 6.87; 95% CI: 2.31–22.27) compared to those with no use. Additionally, one unit increases in HIV stigma (OR = 1.24; 95% CI: 1.01–1.55), everyday discrimination (OR = 1.19; 95% CI: 1.10–1.30), and major forms of discrimination (OR = 1.75; 95% CI: 1.33–2.39) were associated with increases in odds of resource insecurity (Table 3).

**Table 3:** Unadjusted odds ratios for resource insecurity among older people living with HIV (*N* = 52)

Variable	Odds ratio	95 CI
Age	1.14	0.88–1.47
Years living with HIV	0.98	0.72–1.32
Education		
HS degree or less		
Technical school or higher	1.04	0.37–2.89
Employment status		
Full time	Ref	
Part time	**0.19**	**0.05**–**0.67**
Retired	**0.01**	**0.001**–**0.06**
Yearly income – ($25,000 + versus < $25,000)	0.47	0.17–1.31
Sexual orientation (sexual minority versus heterosexual)	**11.64**	**3.55**–**43.92**
History of injection drug use (yes versus no)	**5.62**	**1.93**–**17.62**
History of being unhoused (yes versus no)	1.24	0.45–3.49
History of incarceration (yes versus no)	**3.85**	**1.36**–**11.53**
Cocaine use in the past 3 months (yes versus no)	**6.92**	**2.31**–**22.60**
Opiate use in the past 3 months (yes versus no)	**5.54**	**1.91**–**17.30**
Cannabis use in the past 3 months (yes versus no)	**12.09**	**3.49**–**49.15**
Amphetamine use in past 3 months (yes versus no)	**6.87**	**2.31**–**22.27**
Risky alcohol use (yes versus no)	**8.23**	**1.69**–**61.23**
Overall HIV stigma scale	**1.24**	**1.01**–**1.55**
Personalized stigma (enacted stigma)	**2.33**	**1.27**–**4.81**
Disclosure (anticipated stigma)	1.34	0.67–2.82
Negative self-image (internalized stigma)	0.86	0.60–1.21
Public attitudes (anticipated stigma)	**2.63**	**1.52**–**5.09**
Everyday discrimination scale	**1.19**	**1.10**–**1.30**
Major forms of discrimination scale	**1.75**	**1.33**–**2.39**

*CI*, confidence interval

Bolded numbers significant odds ratios

## Discussion

The aim of this study was to explore the relationship between demographics, social and behavioral factors, and resource insecurity among older AA PWH in Ohio. Study participants were found to have a history of being unhoused, housing instability (lack of stable housing or constant fear of losing it), food insecurity, or a combination of food and housing insecurity. Over one-third of participants self-reported being food and housing insecure. The results of our study showed a number of social and behavioral factors associated with resource insecurity. Resource insecurity was associated with sexual minority identity, history of injection drug use, current substance use, HIV related stigma, and experiences of discrimination.

Our research supports existing literature on the relationship between resource insecurity and social and behavioral factors among PWH. Researchers have shown that compared to White PWH, Black or AA PWH are more likely to experience resource insecurity [35, 36]. We add to the current literature by focusing on older (ages 50 and over) AA males with HIV, who significantly encounter discrimination, and are more likely to experience social determinants of health on the basis of their race [37–39]. To date, the research on resource insecurity among older PWH is limited with little focus on populations most impacted by both HIV and resource insecurity (i.e., older Black PWH) [26]. Additionally, we assessed both food and housing insecurity, and showed how specific illicit drugs were associated with resource insecurity as opposed to any substance use. The ordinal logistic regression results suggest associations between demographics, HIV-related stigma and subtypes, forms of discrimination, illicit substance use, and resource insecurity.

Individuals experiencing food and housing insecurity in the study displayed a higher likelihood of previous incarceration compared to those facing other forms of resource insecurity. Research indicates that individuals with a history of incarceration face a heightened risk of food insecurity, often stemming from inadequate income due to difficulties in securing stable employment [40]. A cross-sectional study by Jordan et al. revealed that a history of incarceration was linked to an increased likelihood of food insecurity [41]. While existing literature does not thoroughly explore the connection between incarceration history and housing instability, some studies suggest a trend of homelessness among individuals recently released from prison [42]. Overall, a history of incarceration may significantly impact the ability of PWH to access food and housing.

Interestingly, those with food and housing insecurity were more likely to have self-reported full-time employment compared to food or housing insecurity and neither. Studies show increased engagement of transactional sex as a method to acquire food or money among PWH with severe food insecurity, creating a vicious cycle [10, 43]. Although the majority of the study group population reported being employed, a significant 59.6% indicated an annual income of less than $25,000, which falls below the poverty line for a family of three [44]. Poverty stands as a robust determinant of health within and across populations. Socioeconomic status plays a crucial role in determining when, if, and what type of healthcare an individual can access and pursue [45]. Even among those who are employed, their income may not suffice to cover all necessary expenses for basic living. PWH are at a heightened risk of poverty, as HIV has been characterized as a disease associated with poverty. Ali [46] describes HIV/AIDS as a disease of poverty because it often results in a lack of choices, leading to an inability to make necessary health decisions that could improve outcomes.

Participants who identified as sexual minorities were at greater odds of being food and housing insecure compared to their heterosexual counterparts. This finding corroborates other studies that have shown sexual minorities are more likely to experience food and housing insecurity compared to their heterosexual counterparts [26, 47]. Stigma and discrimination due to sexual orientation may be driving this finding. Experiences of discrimination based on sexual orientation can lead to financial insecurity which may impact the ability to be housing and food secure. Institutionally, people who identity as a sexual minority may be discriminate against and denied housing as there are no protections against this act. However, participants in our study are living at the intersection of living with HIV, identifying as Black, and identifying as a sexual minority. These identities can intersect to have a larger impact on being food and housing insecure. More research is needed to understand how these multiple identities intersect to impact resource insecurity.

Additionally, the use of recreational or illicit drugs was associated with the experience of food, housing, or resource insecurity among PWH. Specifically, a history of intravenous drug use, as well as the use of cocaine, opiates, cannabis, and amphetamines, along with risky alcohol consumption, were all associated with increased odds of resource insecurity. Resource insecurity, encompassing both food and housing instability, along with substance use, have been consistently linked to suboptimal adherence to HIV care, heightened HIV transmission risk behaviors, and increased morbidity and mortality among PWH [48–50]. In one study, researchers suggested that food insecurity served as a structural driver of substance use. Researchers examined the association between food insecurity and substance use within a cohort of women with or at risk for HIV. Their analysis revealed that women facing the most severe form of food insecurity (termed “very low food security”) exhibited significantly higher odds of engaging in substance use, with a dose-dependent relationship observed. This suggests that women experiencing greater levels of food insecurity are more likely to be involved in substance use, possibly as a coping mechanism to alleviate hunger [17].

Instances of everyday or major discrimination were also associated with greater odds of food or housing insecurity, as well as an elevated odds of resource insecurity (i.e., food and housing). HIV disease burden and mortality is greatest among racial minorities and the poor. In general, there is a low prevalence of HIV in the USA; however, HIV is more concentrated in areas where marginalized communities reside [41, 51]. The intricate interrelationship between discrimination, socioeconomic status, and health has been well-documented in the literature [37, 38]. Racism imposes an additional weight on minority populations, where discrimination and stigma negatively affect health by limiting socioeconomic mobility [37]. A qualitative study in the Dominican Republic found that discrimination both formal and informal, HIV-related stigma and intimate partner violence significantly contributed to food insecurity among women with HIV [39].

Lastly, various aspects of HIV stigma, including overall stigma, personalized stigma (enacted stigma), disclosure-related stigma (anticipated stigma), and public attitudes (anticipated stigma), were associated with increased odds of experiencing resource insecurity. These findings are supported by previous studies indicating that factors such as HIV stigma and discrimination are associated with resource insecurity and further negatively impact health outcomes, specifically among ethnic minorities with HIV [43, 51–53]. Intersectional stigma, defined as stigma from diverse aspects of life, such as race, income, gender, intensifies internalized HIV stigma [11]. Palar et al. [19] suggested severe food insecurity is associated with greater internalized stigma and depressive symptoms among women living with HIV. Interestingly, our results showed no association between internalized stigma and resource insecurity. Furthermore, HIV-positive women who experience food insecurity are more likely to have higher HIV viral loads and lower CD4 counts, highlighting the relationship with food insecurity [52].

## Limitations and Future Directions

This study had several limitations. First, this is the continuation of a pilot study with the goal of exploring the characteristics of PWH and their experience with food and housing insecurity. Our small sample size may not be representative of all older AA PWH in Ohio – the sample was predominately men which did not allow the opportunity to assess differences by sex and/or gender. Secondly, since our data was self-reported, rather than gathered objectively, some individuals may have not felt comfortable to disclose certain information. This may have underestimated the data. Additionally, given the small sample size, we did an unadjusted analysis to explore associations. The study was cross-sectional which does not allow us to make causal claims. Another limitation may be how participants were recruited to participate in the study. Social media was used as the main form of recruitment. This could lead to selection bias due to it restricting potential participants who may not have had access to a computer or phone. Additionally, some individuals facing food and/or housing insecurity may not have access to the tools needed to complete the survey. This potential bias may have led to an underestimate of food and housing insecurity, and thus results must be interpreted with caution. Although lack of transportation accessibility may be considered a resource insecurity, it was not included in this study because in the survey we did not directly ask if participants experienced transportation barriers. Rather, we asked what their main mode of transportation was to make it to the clinic. Further research should be conducted to include transportation as a part of resource insecurity.

Now that we have gained insight on the trends of resource insecurity with demographics and characteristics among older AA PWH, the next step in our study is to explore how resource insecurity relates to HIV outcomes, such as medical adherence and visit adherence. This approach will address critical research gaps by providing valuable insights for comprehensive and targeted interventions.

## Conclusion

Our findings suggest that there is a complex relationship and contributing factors to resource insecurity among the older AA PWH. It is essential to continue these investigations in older AA PWH imperative for mitigating health disparities in HIV.

## Supplementary Information

Below is the link to the electronic supplementary material.

Supplementary file1 (DOCX 701 KB)

## Data Availability

Data may be made available upon request.
