# Supplementary material for: Exploring Correlates of Resource Insecurity Among Older Black or African Americans with HIV in Ohio
Source: J Racial Ethn Health Disparities. 2024 Sep 4;12(6):3576–86. doi: 10.1007/s40615-024-02158-y (PMC12644116; doi:10.1007/s40615-024-02158-y)
Supplement: Supplementary file 1 — Supplementary file1 (DOCX 701 KB) [file 40615_2024_2158_MOESM1_ESM.docx]

Online Resource 1 – Full questionnaire of the online survey.

**Journal of Racial and Ethnic Health Disparities**

**Exploring Correlates of Resource Insecurity Among Older Black or African Americans with HIV in Ohio**

Yanil V. Ramirez^1^, Gisella M. Drouet Saltos^1^, Timothy N. Crawford^1,2,3^

^1^Wright State University Boonshoft School of Medicine, Dayton, OH, USA

^2^Wright State University Boonshoft School of Medicine, Population and Public Health Sciences, Dayton, OH, USA

^3^Wright State University Boonshoft School of Medicine, Family Medicine, Dayton, OH, USA

Corresponding Author

Timothy N. Crawford

[Timothy.crawford@wright.edu](mailto:Timothy.crawford@wright.edu)

ORCID: **0000-0001-8005-4495**

**We are interested in the social and emotional aspects of having HIV. There are no right or wrong answers. Feel free to write in comments as you go through the questions.**

**This first set of questions asks about some of your experiences, feelings, and opinions about how people with HIV feel and how they are treated. Please try to answer each question.**

**For each item, click on your answer. Your choices are: Strongly Disagree (SD), Disagree (D), Agree (A), or Strongly Agree (SA)**

I feel I am not as good a person as others because I have HIV

Having HIV makes me feel unclean

Most people think that a person with HIV is disgusting

Having HIV makes me feel that I am a bad person

Most people with HIV are rejected when others find out

I am very careful who I tell that I have HIV

Strongly Disagree

(SD)


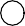

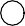

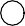

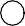

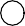

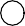


Disagree (D) Agree (A) Strongly Agree (SA)


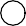

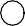

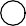

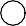

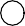

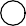

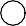

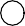

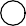

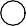

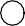

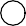

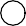

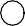

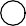

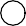

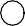

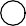


**Many of the items in this next section assume that you have told other people that you have HIV, or that others know. This may not be true for you. If the item refers to something that has not actually happened to you, please imagine yourself in that situation. If this is the case, please give your answer based on how you think you would feel or how you thing others would react to you. Your choices are: Strongly Disagree (SD), Disagree (D), Agree (A), or Strongly**

**Agree (SA).**

I have been hurt by how people reacted to learning I have HIV

I worry that people who know I have HIV will tell others

I have stopped socializing with some people because of their reactions to my having HIV

Strongly Disagree

(SD)


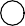

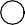

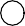


Disagree (D) Agree (A) Strongly Agree (SA)


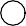

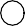

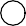

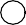

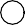

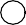

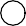

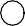

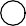


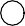

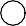

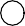

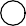
I have lost friends by telling them I have HIV

**In your day-to-day life, how often do any of the following things happen to you?**

You are treated with less courtesy than other people are

You are treated with less respect than other people are

You receive poorer service than other people at restaurants or stores

Almost

everyday


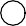

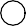

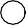


At least once

a week


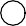

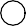

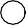


A few times a

month


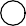

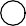

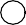


A few times a

year


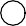

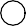

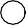


Less than

once a year


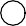

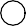

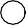


Never


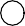

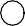

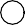


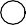

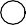

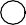

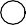

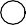

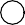
People act as if they think you are not smart


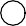

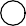

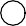

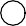

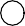
People act as if they are afraid of
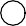
 you


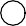

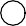

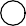

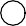

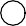

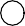
People act as if they think you are dishonest


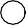

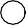

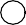

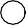

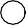

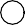
People act as if they're better than you are


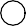

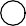

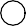

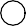

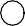

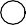

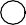

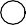

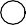

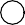
You are called names or insults
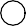
 You are threatened or harassed
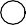


What do you think is the main reason for these
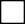
 Your Ancestry or National Origins

experiences? CHECK ALL THAT APPLY
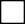
 Your Gender
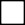
 Your Race
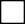
 Your Age


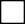
 Your Religion
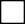
 Your Height Your Weight

Some other Aspect of Your Physical Appearance Your Sexual Orientation

Your Education or Income Level

**In the following questions, we are interested in the way other people have treated you or your**

**beliefs about how other people have treated you. Can you tell me if any of the following has ever happened to you:**

Yes No

At any time in your life, have you ever not been hired for a job?

For unfair reasons, have you ever not been hired for a job?

Have you ever been unfairly denied a promotion?

Have you ever been unfairly stopped, searched, questioned, physically threatened or abused by the police?

Have you ever been unfairly discouraged by a teacher or advisor from continuing your edcuation?

Have you ever been unfairly prevented from moving into a neighborhood because the landlord or a realtor refused to sell or rent you a house or apartment?

Have you ever moved into a neighborhood where neighbors made life difficult for you or your family?

Have you ever been unfairly denied a bank loan?

Have you ever received service from someone such as a plumber or car mechanic that was worse than what other people get?

What do you think was the main reason for this Your Ancestry or National Origins

experience? Your Gender

Your Race Your Age

Your Religion Your Height Your Weight

Some other Aspect of Your Physical Appearance Your Sexual Orientation

Your Education or Income Level

When was the last time this happened? Past week Past month Past year

More than a year ago

How many times has this happened during your lifetime?

**These questions are about your feelings and thoughts during the last month. For each item, please indicate by selecting how often you felt or thought a certain way. Your choices are:**

**0 = Never**

**1 = Almost Never**

**2 = Sometimes**

**3 = Fairly Often**

**4 = Very Often**

1. In the last month, how often have you been upset 0 = Never because of something that happened unexpectedly? 1 = Almost Never

2 = Sometimes

3 = Fairly Often

4 = Very Often

1. In the last month, how often have you felt that you 0 = Never

were unable to control the important things in your 1 = Almost Never life? 2 = Sometimes

3 = Fairly Often

4 = Very Often

1. In the last month, how often have you felt nervous 0 = Never

and "stressed"? 1 = Almost Never

2 = Sometimes

3 = Fairly Often

4 = Very Often

1. In the last month, how often have you felt 0 = Never confident about your ability to handle your personal 1 = Almost Never problems? 2 = Sometimes

3 = Fairly Often

4 = Very Often

1. In the last month, how often have you felt that 0 = Never

things were going your way? 1 = Almost Never

2 = Sometimes

3 = Fairly Often

4 = Very Often

1. In the last month, how often have you found that 0 = Never

you could not cope with all the things that you had to 1 = Almost Never do? 2 = Sometimes

3 = Fairly Often

4 = Very Often

1. In the last month, how often have you been able to 0 = Never control irritations in your life? 1 = Almost Never

2 = Sometimes

3 = Fairly Often

4 = Very Often

1. In the last month, how often have you felt that you 0 = Never

were on top of things? 1 = Almost Never

2 = Sometimes

3 = Fairly Often

4 = Very Often

1. In the last month, how often have you been angered 0 = Never because of things that were outside of your control? 1 = Almost Never

2 = Sometimes

3 = Fairly Often

4 = Very Often

1. In the last month, how often have you felt 0 = Never difficulties were piling up so high that you could not 1 = Almost Never overcome them? 2 = Sometimes

3 = Fairly Often

4 = Very Often

**Brief Resilience Scale**

I tend to bounce back quickly after hard times. Strongly Disagree Disagree

Neutral Agree

Strongly Agree

I have a hard time making it through stressful events. Strongly Disagree

Disagree Neutral Agree

Strongly Agree

It does not take me long to recover from a stressful Strongly Disagree event. Disagree

Neutral Agree

Strongly Agree

It is hard for me to snap back when something bad Strongly Disagree

happens. Disagree

Neutral Agree

Strongly Agree

I usually come through difficult times with little Strongly Disagree

trouble. Disagree

Neutral Agree

Strongly Agree

I tend to take a long time to get over set-backs in my Strongly Disagree life. Disagree

Neutral Agree

Strongly Agree

**Over the last 2 weeks, how often have you been bothered by any of the following problems?**

Little interest or pleasure in doing things

Feeling down, depressed, or hopeless

Trouble falling or staying asleep, or sleeping too much

Feeling tired or having little energy

Poor appetite or overeating

Feeling bad about yourself or that you are a failure or have let yourself or your family down

Not at all Several days More than half the

days

Nearly every day

Trouble concentrating on things, such as reading the newspaper or watching television

Moving or speaking so slowly that other people could have noticed. Or the opposite - being so figety or restless that you have been moving around a lot more than usual

**People sometimes look to others for companionship, assistance, or other types of support.**

**How often is each of the following kinds of support available to you if you need it?**

Someone to help with daily chores if you were sick

Someone to turn to for suggestions about how to deal with a personal problem

None of the time A little of the

time

Some of the time Most of the time All of the time

Someone to do something enjoyable with

Someone who understands your problems

**In this section, there are a number of statements with which you may or may not agree. For each statement listed, please indicate whether you personally agree or disagree with it using a scale where 1 means "strongly disagree," 2 means "somewhat disagree," 3 means "neither agree nor disagree," 4 means "somewhat agree," and 5 means "strongly agree." If you don't**

**understand a statement or it is not applicable to you, please leave that row blank.**

I am satisfied with my life

My life has a clear sense of purpose

Most days I feel a sense of accomplishment from what I do

Strongly

disagree

2 3 4 Strongly agree

**How much of the time during the past 30 days have you felt...?**

None of the time 2 Some of the time 4 All of the time

Cheerful Hopeless

Your family life

**Please tell me on a scale of 1 to 10 how satisfied you are with each of the following items, where 1 means "very dissatisfied" and 10 means "very satisfied."**

Your friends and social life Your energy level

Very Dissatis fied

2 3 4 5 6 7 8 9 Very

Satisfie d

In general, would you say your health is...? Excellent Very good Good

Fair Poor

During the past 30 days, for about how many days have 0-5 days you felt very healthy and full of energy? 6-11 days

12-17 days

18-23 days

24-30 days

**In your life, which of the following substances have you ever used?**

No Yes

1. Tobacco products (cigarettes, chewing tobacco, cigars, etc.)
2. Alcoholic beverages (beer, wine, spirits, etc.)
3. Cannabis (marijuana, pot, grass, hash, etc.)
4. Cocaine (coke, crack, etc.)
5. Amphetamine type stimulants (speed, diet pills, ecstasy, etc.)
6. Inhalants (nitrous, glue, petrol, paint thinner, etc.)
7. Sedatives or Sleeping Pills (Vallium, Serepax, Rohypnol, etc.)
8. Hallucinogens (LSD, acid,

mushrooms, PCP, Special K, etc.)

1. Opioids (heroin, morphine, methadone, codeine, etc.)
2. Other

**In the past three months, how often have you used the substances you mentioned (FIRST**

**DRUG, SECOND DRUG, ETC)?**

1. Tobacco products (cigarettes, chewing tobacco, cigars, etc.)
2. Alcoholic beverages (beer, wine, spirits, etc.)
3. Cannabis (marijuana, pot, grass, hash, etc.)
4. Cocaine (coke, crack, etc.)
5. Amphetamine type stimulants (speed, diet pills, ecstasy, etc.)
6. Inhalants (nitrous, glue, petrol, paint thinner, etc.)
7. Sedatives or Sleeping Pills (Vallium, Serepax, Rohypnol, etc.)
8. Hallucinogens (LSD, acid,

mushrooms, PCP, Special K, etc.)

1. Opioids (heroin, morphine, methadone, codeine, etc.)
2. Other

Never Once or Twice Monthly Weekly Daily or Almost

Daily

How often do you have a drink containing alcohol? Never

Monthly or less

2-4 times a month 2-3 times a week

4 or more times a week

How many drinks containing alcohol do you have on a 1 or 2 typical day when you are drinking? 3 or 4 5 or 6

7 to 9

10 or more

How often do you have five or more drinks on one Never

occasion? Less than monthly

Monthly Weekly

Daily or almost daily

In the past four weeks, did you worry that your Yes

household would not have enough food? No

How often did this happen? Rarely (once or twice in the past four weeks) Sometimes (three to ten times in the past four weeks)

Often (more than ten times in the past four weeks)

In the past four weeks, were you or any household Yes

member not able to eat the kinds of foods you No preferred because of a lack of resources?

How often did this happen? Rarely (once or twice in the past four weeks) Sometimes (three to ten times in the past four weeks)

Often (more than ten times in the past four weeks)

In the past four weeks, did you or any household Yes member have to eat a limited variety of foods due to a No lack of resources?

How often did this happen? Rarely (once or twice in the past four weeks) Sometimes (three to ten times in the past four weeks)

Often (more than ten times in the past four weeks)

In the past four weeks, did you or any household Yes member have to eat some foods that you really dis not No want to eat because of a lack of resources to obtain

other types of food?

How often did this happen? Rarely (once or twice in the past four weeks) Sometimes (three to ten times in the past four weeks)

Often (more than ten times in the past four weeks)

In the past four weeks, did you or any household Yes

member have to eat a smaller meal than you felt you No needed because there was not enough food?

How often did this happen? Rarely (once or twice in the past four weeks) Sometimes (three to ten times in the past four weeks)

Often (more than ten times in the past four weeks)

In the past four weeks, did you or any other household Yes member have to eat fewer meals in a day because there No was not enough food?

How often did this happen? Rarely (once or twice in the past four weeks) Sometimes (three to ten times in the past four weeks)

Often (more than ten times in the past four weeks)

In the past four weeks, was there ever no food to eat Yes

of any kind in your household because of lack of No resources to get food?

How often did this happen? Rarely (once or twice in the past four weeks) Sometimes (three to ten times in the past four weeks)

Often (more than ten times in the past four weeks)

In the past four weeks, did you or any household Yes member go to sleep at night hungry because there was No not enough food?

How often did this happen? Rarely (once or twice in the past four weeks) Sometimes (three to ten times in the past four weeks)

Often (more than ten times in the past four weeks)

In the past four weeks, did you or any household Yes

member go a whole day and night without eating No anything because there was not enough food?

How often did this happen? Rarely (once or twice in the past four weeks) Sometimes (three to ten times in the past four weeks)

Often (more than ten times in the past four weeks)

**Your primary health care provider is probably your doctor. However, your health care provider might also be a nurse practitioner or physician's assistant. Please check the type of health care provider that you see on a regular basis and then complete the following questions with**

**that person in mind.**

My primary health care provider is (please check one): Doctor

Nurse Practitioner Physician Assistant Other

Other, please describe

**Please rate the degree to which each statement reflects your provider's behavior toward you on the following scale:**

**1 = Always**

**2 = Usually**

**3 = Sometimes**

**4 = Never**

**N/A = No Experience**

**My health care provider:**

Always Usually Sometimes Never N/A

Listens to me Cares about me

Answers my questions Spends enough time with me Involves me in decisions Respects my choices

Deals with my problems Engages me in my care Is helpful to me Respects me

Supports my decisions Sees me when I ask

Provides me with information

**This section will ask about your HIV clinical characteristics.**

What year were you diagnosed with HIV?

In what year did you seek HIV care?

Are you currently seeking HIV care? Yes No

Where are you seeking HIV care?

What was your possible HIV transmission route? Sex with a male Sex with a female

Heterosexual transmission

Men who have sex with men (MSM)

Injection Drug Use (Sharing needles, syringes or other injection equipment with infected person) Mother-to-child transmission

Transfusions Occupational exposure

Sex with a male and Injection Drug Use Other

Other, please specify

Have you ever been diagnosed with AIDS? Yes No

Are you currently taking any HIV medication? Yes No

In what year did you start HIV medication?

Thinking about the past 4 weeks, on average how would Very poor you rate your ability to take all of your HIV Poor

antiretroviral medications as your doctor prescribed? Fair Good

Very good Excellent

On a scale from 0 to 10, 0 being never, and 10 being Never

always, how would you rate yourself in terms of 1

staying on track with taking your antiretrovirals? 2

3

4

5

6

7

8

9

Always

Thinking back over the past 3 months, has there ever Yes

been a time where you missed taking all of your No antiretrovirals for 4 days or more?

Do you know your current CD4 cell count? Yes No

What is your current CD4 cell count?

Do you know your current viral load? Yes No

What is your current viral load? Detectable (≥ 40 copies/ml) Undetectable (< 40 copies/ml)

Since seeking HIV care, how many times have you fallen out of care (not receiving HIV care for 12 or more months)?

In the past 12 months, did you miss any HIV clinic Yes appointments (Does not include appointments canceled No and rescheduled)?

How many HIV clinic appointments did you miss?

In the past 6 months, did you miss any clinic Yes appointments (Does not include appointments canceled No and rescheduled)?

How many clinic appointments did you miss?

What is your PRIMARY mode of transport to HIV clinic Own car appointments? Borrow car

Get ride

Public Transportation (e.g., bus) Ride share (e.g., Uber, Lyft) Walk

How many miles is it from your home to the HIV clinic?

In minutes, how long does it take to get from home to the HIV clinic?

Have you ever being diagnosed with any of the Alcohol Dependence

following conditions? (Check all that apply) Anxiety Arthritis Asthma Cancer

Chronic Obstructive Pulmonary Disorder (COPD) Coronary Artery Disease

Diabetes Depression hepatitis B hepatitis C

Hypertension Renal Disease Stroke

Other

Other, please specify

**This final section will ask about your demographic characteristics.**

What is your age?

Do you identify as transgender? Yes No

What gender do you most identify with? Female Male Genderqueer/Gender fluid Non-binary Another Identity

Another Identity, Please Specify

What is your sexual orientation? Asexual Bisexual

Gay Lesbian Pansexual Queer Straight (heterosexual) Another Identity

Another Identity, Please Specify

Have you ever had sex with a man? Yes No

Have you ever had sex with a woman? Yes No

What is the highest educational level you completed? Less than high school

High School/GED Some College Technical School Associate's Degree Bachelor's Degree Master's Degree Doctoral Degree

What is your employment status? Full time Part time Unemployed

Unable to work Retired

Other

Other, please specify

What is your annual household income?

What is your monthly household income?

Which of the following best describes the residence in House that you own

which you currently live? Apartment or condominium that you own House that you rent

Floor in a house that you rent

A basement apartment that you rent Apartment or condominium that you rent

Self-contained room in a house with other people Self-contained room in an apartment with other people

Self-contained room with amenities Self-contained room with no amenities HIV care group home

A housing facility

outdoors/on the street/parks/in a car Couch surfing

Transition house/halfway house/safe house Shelter

Jail

Have you ever been without a permanent address you Yes call home? No

Given your total household income, how difficult is it to meet your monthly housing costs including rent/mortgage, property taxes, and utilities (e.g., heat, electricity, water, and gas)?

Very Easy Fairly Easy Neutral Fairly Difficult Very Difficult

Have you ever been incarcerated? Yes No

Were you diagnosed with HIV while incarcerated? Yes No

Have you ever injected drugs? Yes

No

What kind of insurance do you have? (Check multiple if Private

needed) Ryan White Care Act

Medicaid Medicare Other

Other, please specify

# Intersectional Stigma and HIV Engagement in Care Follow-up

Please answer the questions below. Thank you!

Thank you for completing the survey. In order for us

to send you your $25 gift card, we will nee a valid email address. Note: this identifying information will

not be tied to your survey responses.

The study has two phases. The first phase was Yes

completion of the survey/questionnaire. The second No phase involves a more in-depth interview to understand

how your experiences impacts engagement in HIV care.

Would you be willing to participate in the second phase of the study?

Please provide a phone number where we can contact you

to set up an appointment to complete the second phase of the study.

Please provide an email address where we can contact

you to set up an appointment to complete the second phase of the study.
